# Why acute unilateral vestibular midbrain lesions rarely manifest with rotational vertigo: a clinical and modelling approach to head direction cell function

**DOI:** 10.1007/s00415-018-8828-5

**Published:** 2018-03-16

**Authors:** Marianne Dieterich, Stefan Glasauer, Thomas Brandt

**Affiliations:** 1 Department of Neurology, University Hospital, Ludwig-Maximilians Universität München, Marchioninistrasse 15, 81377 Munich, Germany; 2 German Center for Vertigo and Balance Disorders, Ludwig-Maximilians Universität, Munich, Germany; 3 Clinical Neuroscience, Ludwig-Maximilians Universität, Munich, Germany; 4 Munich Cluster for Systems Neurology (SyNergy), Munich, Germany

**Keywords:** Midbrain stroke, Rotational vertigo, Unspecific dizziness, Vestibular system, Head-angular velocity cells, Head direction cells, Mathematical model

## Abstract

A retrospective clinical study focused on the frequency of rotational vertigo in 63 patients with acute unilateral midbrain strokes involving the vestibular and ocular motor systems. In contrast to unilateral pontomedullary brainstem lesions, rotational vertigo in midbrain lesions occurred with a low frequency (14%) and transient (< 1 day) course. Swaying vertigo or unspecific dizziness (22%) and postural imbalance (31%) were more frequent. Midbrain strokes with transient rotational vertigo manifested with lesions chiefly in the caudal midbrain tegmentum, while manifestations with swaying, unspecific, or no vertigo chiefly occurred in rostral mesencephalic or meso-diencephalic lesions. We hypothesize that these different manifestations can be explained by the distribution of two separate cell systems based on semicircular canal function: the angular head-velocity cells and the head direction cells, both of which code for head rotation. Animal experiments have shown that angular head-velocity cells are located mainly in the lower brainstem up to the midbrain, whereas the head direction cells are found from the midbrain and thalamic level up to cortical regions. Due to the differences in coding, unilateral dysfunction of the angular velocity cell system should result in the sensation of rotation, while unilateral dysfunction of the head direction cell system should result in dizziness and unsteadiness. We simulated the different manifestations of vestibular dysfunction using a mathematical neural network model of the head direction cell system. This model predicted and confirmed our clinical findings that unilateral caudal and rostral brainstem lesions have different effects on vestibular function.

## Introduction

**Fig. 1:**
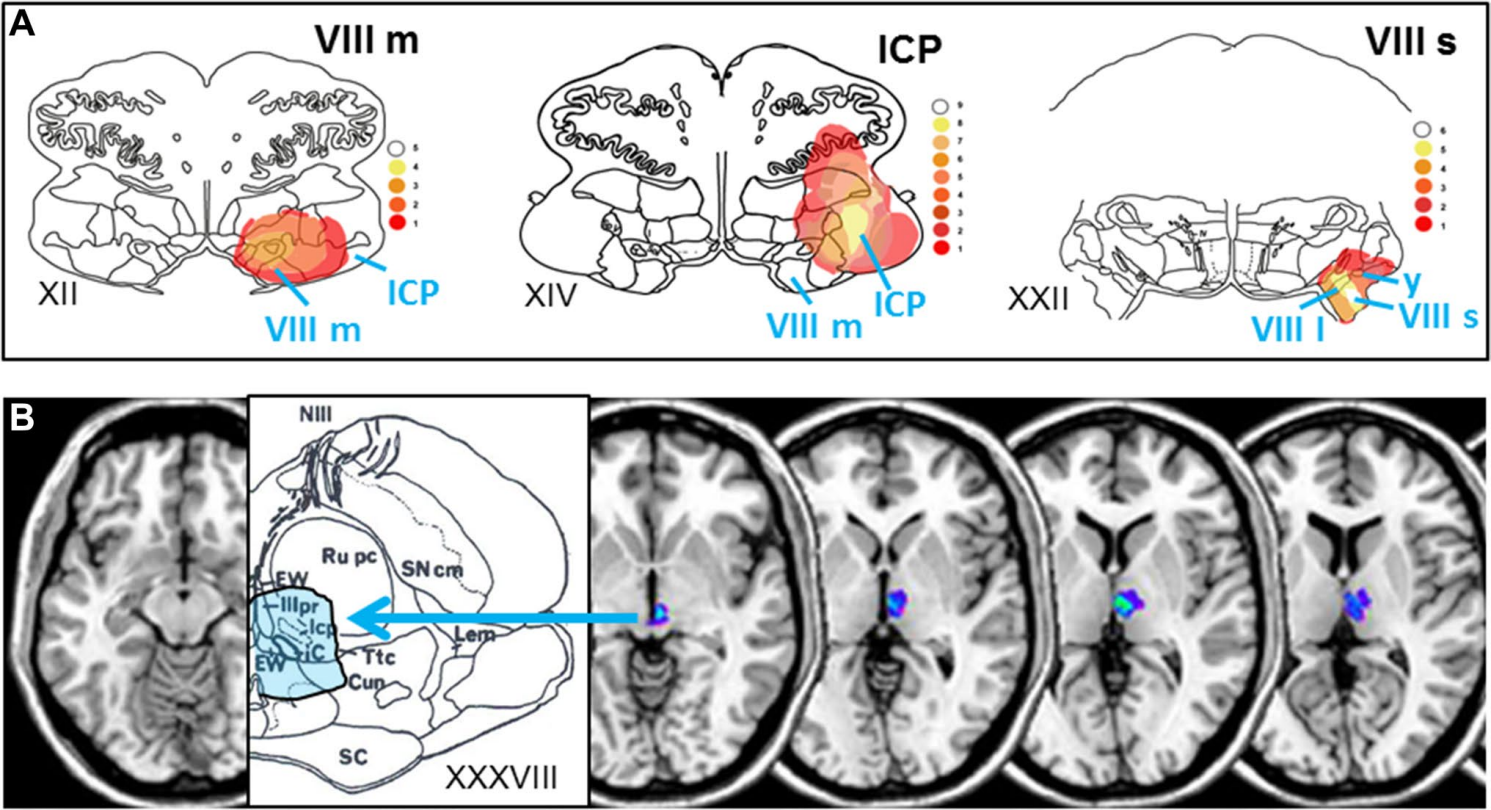
Brainstem lesions causing an acute vestibular syndrome. **a** Overlap areas of brainstem infarcts in 23 patients who presented with an isolated vestibular syndrome with acute sustained rotational vertigo that mimicked an acute unilateral peripheral vestibulopathy. MRI data from the literature [3–5, 7, 10] were superimposed on three sections (XII, XIV, XXII) of the human brainstem atlas of Olszewski and Baxter [61] (methods described in [27]). Overlap areas involve chiefly the medial vestibular nucleus (VIII m), the inferior cerebellar peduncle (ICP), the superior and lateral vestibular nucleus (VIII s, VIII l) and the y-group (y, small cell group in the dorsolateral pontine tegmentum). (modified from [31]). **b** Overlap areas of rostral mesencephalic–diencephalic lesions in eight patients with acute unilateral midbrain infarctions affecting the interstitial nucleus of Cajal (iC) and the rostral interstitial nucleus of the medial longitudinal fascicle (riMLF; not shown on the magnified slice XXXVIII of the brainstem atlas of Olszewski and Baxter). In all 8 patients the strokes caused a sustained instability of stance and gait with skew torsion of the eyes. Only one patient reported an initially transient rotatory vertigo, two a swaying vertigo, and five no vertigo/dizziness at all (modified from [17]). *EW* Edinger–Westphal nucleus, *icp* nucleus intracapsularis, *IIIpr* nucleus oculomotorius principalis

Acute unilateral peripheral vestibular syndromes that involve semicircular canal function and are caused by a labyrinthine (Meniere’s disease) or vestibular nerve (acute vestibular syndrome, AVS) disorder manifest with rotational vertigo and horizontal-rotatory spontaneous nystagmus. Pathophysiologically, it is well acknowledged that these signs and symptoms are caused by a vestibular tone imbalance generated by unequal input from the right and left ears. The side of the unilateral lesion that causes the tone imbalance can be determined by the direction of vertigo and spontaneous nystagmus, which is contralateral and by the deviation of stance and gait, which is ipsilateral to the lesion side. Similar signs and symptoms occur with unilateral vestibular lesions of the root entry zone of the eighth nerve affecting the vestibular fascicle, the vestibular nucleus (chiefly medial and superior parts) [1–7], the nucleus prepositus hypoglossi [7], the cerebellar peduncle [7, 8], and the vestibular cerebellum [8–13]. As these syndromes are elicited by central rather than peripheral vestibular structures, they are called central vestibular pseudoneuritis [14, 15] or, more recently, central acute vestibular syndrome [16]. It is noteworthy that the latter central lesions are all located at the lower brainstem and cerebellar levels. In Fig. 1a, we have depicted the overlap of causative MRI lesions at pontomedullary level in 23 stroke patients reported in the literature who presented with sustained rotational vertigo [3–5, 7, 10] (Fig. 1a).

The clinical manifestation of more cranial unilateral vestibular lesions in the meso-diencephalic brainstem and the vestibular cortex differs. In a small case study of eight patients with acute unilateral lesions of the midbrain, only one patient initially had rotational vertigo and two, swaying vertigo [17] (Fig. 1b). Furthermore, unilateral lesions of the vestibular thalamic subnuclei (posterolateral and centromedian) do not manifest with rotational vertigo but rather with thalamic astasia [18–20] and tilts of perceived visual vertical [21–23]. Acute vestibular cortex lesions caused by strokes in the middle cerebral artery territory present with transient vertigo only in exceptional cases [24–27].

The discrepancy of clinical manifestations with rotational vertigo in peripheral and central vestibular lesions of the caudal brainstem and without rotational vertigo in meso-diencephalic and vestibular cortex lesions raises the question of whether specialized motion-detecting cell systems that mediate angular velocity and others that mediate spatial orientation and head direction are differently involved.

We hypothesize that the rare or absent condition of rotational vertigo in patients with thalamic and cortical strokes can be explained by the change that takes place in the neuronal coding of vestibular motion signals on their way from the vestibular labyrinthine receptors in the semicircular canals and vestibular nuclei to the cortex: here, a representation of velocity is transformed into a representation of head position [28–30]. The latter is necessary for an individual to monitor both the rotation and the change of body direction in space due to the rotation. This makes it possible to continuously update the cognitive map of body position and orientation in space [31].

It has been found in rodents that the representation of self-motion, speed and direction, current heading, and location in space is mediated by various neuronal subsystems that encode different features. Location in space is encoded by hippocampal place cells; head rotation by angular head-velocity cells; heading direction by head direction cells; and distance by entorhinal cortex grid cells [32–34]. The neuronal assemblies are anatomically widely distributed and functionally interconnected with the hippocampal formation [34, 35]. Recent experiments on the medial entorhinal cortex have revealed further coding principles with a high degree of mixed selectivity and heterogeneity of single cells as well as adaptive coding for navigational information at varying speeds [36].

**Fig. 2:**
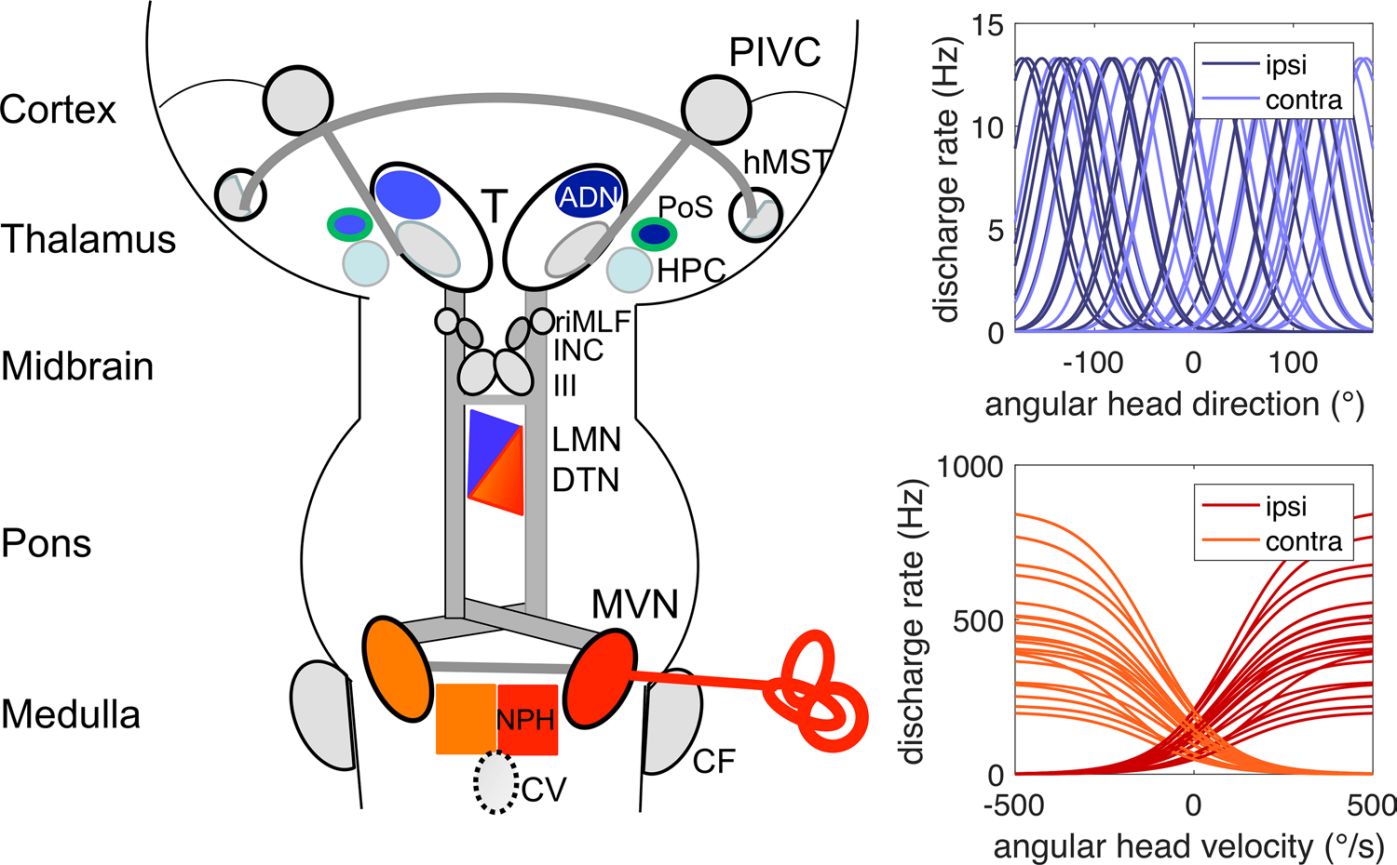
Schematic anatomic pathways (left) and activation functions of neurons (right) involved in angular spatial orientation. Left: schematic drawing of the bilateral structural organization of the vestibular system from the vestibular nuclei (MVN) to multisensory vestibular cortex areas such as the parieto-insular vestibular cortex (PIVC) and the human medial superior temporal area (hMST) of the visual cortex. Vestibular input from the endorgan (red) ascends ipsilaterally and contralaterally mainly via the medial longitudinal fascicle to the midbrain tegmentum with the dorsal tegmental nucleus (DTN) and the lateral mammillary nucleus (LMN), which lie below the ocular motor nuclei complex (III), the interstitial nucleus of Cajal (INC), and the rostral interstitial nucleus of the medial longitudinal fascicle (riMLF). The angular head-velocity cells (red) originate from the semicircular canals and are located within the medial vestibular nucleus (MVN), the nucleus praepositus hypoglossi (NPH), the supragenual nucleus, and the paragigantocellular reticularis nucleus dorsalis (both not depicted). The representation of head-velocity cells is proposed to become less in the upper brainstem, such as the DTN and even less in the LMN [43]. A head direction cell system (blue) is distributed in the upper midbrain and the anterior dorsal thalamus (ADN) and various cortex areas, such as the postsubiculum (PoS), and the retrosplenial cortex (not depicted). Place cells (light blue) are located mainly in the hippocampal formation (HPC); grid cells (green) have been found in the PoS. Vestibular structures are depicted in gray including the cerebellar flocculus (CF) and the cerebellar vermis (CV) which is projected onto the level of the medullary brainstem (stippled). Right: each curve represents the schematic response of a single neuron to its stimulus. Lower right: discharge rate plotted over angular velocity for hypothetical primary afferent canal neurons or angular velocity neurons in the vestibular nuclei or brainstem. Neurons on the ipsilateral side respond with increasing firing rate for ipsilateral turns and vice versa. Upper right: discharge rate plotted over head direction for hypothetical head direction cells. Neurons on ipsi- and contralateral sides of the brain respond similarly being tuned to a specific head direction

Here, we will focus on two cell types, which are closely related to semicircular canal function and to the question of why a unilateral vestibular lesion manifests with rotational vertigo. These are the angular head-velocity cells, which originate from the semicircular canals with a network mainly distributed infratentorially, and the head direction cells, which are mainly distributed in the upper brainstem and in supratentorial areas (Fig. 2). The change of angular neuronal vestibular coding, i.e., from a velocity- to a positional signal, mediated by the two cell systems provides the basis for our current hypothesis.

The clinical approach was to retrospectively analyse patients’ data for cases of acute unilateral midbrain strokes involving vestibular function with a focus on the frequency of rotational vertigo, spontaneous nystagmus, and other forms of dizziness and unsteadiness. The retrospective approach was chosen to gather information from a larger number of patients who underwent standardized neuro-otological work-ups. If the occurrence of rotational vertigo was dependent on a dysfunction of the head-angular velocity cell system, these signs and symptoms should become less frequent with upper mesencephalic lesions, because this particular cell system is topographically located chiefly in the lower brainstem and cerebellum. The clinical study was complemented and further interpreted by an attempt to mathematically model the effects of a unilateral disruption within the head-angular velocity cell system as opposed to a unilateral disruption of the head direction cell system. Computer simulation of the head direction cell network from peripheral vestibular input to cortical levels was based on a previously proposed attractor network approach [37].

## Methods

### Clinical study

#### Patients

A retrospective analysis was made of a total of 158 patients who presented with a midbrain syndrome in the Department of Neurology and the German Center of Vertigo and Balance Disorders, University Hospital of Munich, Ludwig-Maximilians University, Germany, over a 10-year period from January 2007 to May 2017.

Inclusion criteria were acute vestibular and/or ocular motor syndromes due to unilateral midbrain strokes. Exclusion criteria were chronic or slowly progressive midbrain disorders such as progressive supranuclear palsy, cases of earlier brainstem or multiple brain lesions, and strokes with a symmetrical bilateral involvement of the midbrain.

According to these criteria, 63 patients with acute unilateral midbrain strokes (37 left-sided, 26 right-sided) were included. All patients were asked to indicate their vestibular sensations according to one of the four categories: (1) rotational vertigo; (2) swaying vertigo; (3) unspecific dizziness; and (4) postural imbalance and unsteadiness of gait without vertigo. All patients underwent a standardized neurological, neuro-otological, and neuro-ophthalmological work-up performed by specialized physicians and professional orthopticians. The following functions were assessed: clinical head impulse test, rapid and smooth pursuit eye movements, gaze and ocular motor nerve function, spontaneous-, optokinetic- and gaze-evoked nystagmus, spontaneous deviation from normal head upright position, and signs of graviceptive (otolith/vertical semicircular canal) dysfunction. It was also checked for a vestibular tone imbalance in the roll plane, i.e., the components of an ocular tilt reaction (OTR) such as head tilt, skew deviation, ocular torsion, and perception of subjective visual vertical (SVV) [17, 38]. Fundus photography was performed with a laser ophthalmoscope to determine ocular torsion. Adjustments of the SVV were measured binocularly in an upright position, while the patient looked into a hemispheric dome (60 cm in diameter) covered with a random pattern of coloured dots, containing no clues about gravitational orientation. The patient had to adjust a central test target (straight line in the centre of the dome) from a random offset position to the perceived vertical using a potentiometer. SVV is determined by calculating the means of ten adjustments (pathological deviation > 2.5°) [38]. A complete ocular tilt reaction (OTR) was defined as the combination of all three signs of vestibular tone imbalance in the roll plane of the vestibular ocular reflex (i.e., lateral head tilt, skew deviation of the eyes, and ocular torsion) [39].

An MRI of the brain was available for all patients. Evaluations of these MRIs by an experienced neurologist and a neuroradiologist were used to determine the side and the level of the brainstem lesions and the amount of bilaterality as a criterion for inclusion or exclusion as well as proof that disorders other than acute strokes had been excluded.

### Mathematical modelling of central processing of semicircular canal input

We first describe the essential concepts behind the model and then turn to the mathematical description.

#### Vestibular coding of velocity

Turning of the head to the ipsilateral side causes an increase in the firing rate of sensory haircells of the horizontal semicircular canal, while turning to the contralateral side causes a decrease of the firing rate. The firing rate approximately codes for head velocity in a proportional way (for schematic tuning curves, see Fig. 5b). Thus, a head turn to the right would cause a decrease of the firing rate in the left vestibular nerve and an increase in the right nerve, from a resting rate of around 100 Hz in squirrel monkeys [40] and probably more in humans. This type of excitation–inhibition coupling has also been called a push–pull configuration. Such a configuration with rate coding is useful for motor control and is maintained throughout the brainstem circuitry up to the ocular motor nuclei in the midbrain tegmentum, where vestibular input drives the vestibulo-ocular reflex. Both sides of the vestibular nuclei, the first relay station of vestibular afferent information, are densely connected by commissural fibers, which transmit afferent vestibular information from one side to the other, so that sensory information from both sides is fused already at the level of the vestibular nuclei [41]. Rate coding is not only found in the brainstem, but also in the Purkinje cells of the vestibulo-cerebellum, which projects back to target neurons in the vestibular nuclei, for example, gaze-velocity Purkinje cells in the floccular lobe code in push–pull configuration for horizontal gaze velocity [42]. In principle, it would be sufficient to read out one single rate neuron to decode actual head velocity. As shown in rodent studies (see [43] for review), this type of coding continues up to the angular head-velocity cells in the dorsal tegmental nuclei, which code for angular head velocity in the horizontal plane, and constitute an important step towards head direction cells in the lateral mammillary nuclei (LMN) and the antero-dorsal thalamic nuclei (ADN).

#### Vestibular coding of head direction

In contrast to angular head-velocity cells described above, head direction cells are tuned to a specific angular direction in the horizontal plane and can be conceived as an inbuilt compass that indicates a specific direction in allocentric space (relative to the environment), while we move around. A single neuron in the head direction cell system might thus discharge maximally when the head is oriented towards west, would decrease its firing rate when the head is turned away either to the north or south, and would fire minimally for an eastward head direction. Conversely, other head direction cells would fire maximally when the head is oriented to the east, north, or any other direction (for schematic tuning functions, see Fig. 5). Head direction cells have been found throughout the brain, first occurring in the midbrain (LMN) and thalamus (ADN). Cortical regions containing head direction cells include the retrosplenial and entorhinal cortices [43]. It is generally assumed that head direction cells form circular attractor networks [44] due to their reciprocal connectivity. In contrast to single-neuron-based rate coding for angular head velocity, the information about current head direction cannot be decoded from a single neuron; it requires a read-out of several neurons to get an estimate of the population response.

#### Mathematical formulation

On the basis of the above considerations, we modeled the putative pathways and structures from the angular head-velocity inputs to the postsubiculum (PoS) by computer simulation of an extended version of a previously proposed attractor network for head direction cells [37], based on [45, 46]. We chose this model over others (e.g., [47–49]) because of its neurophysiologically plausible but simple structure. For the present purpose, we extended that model by a level representing the thalamic antero-dorsal nuclei (ADN) and the postsubiculum (PoS) (Fig. 5a). Briefly, six populations of firing-rate neurons (100 neurons per population) with a linear-threshold activation function (no negative firing rates) and Gaussian synaptic weight distributions (i.e., weights decrease with functional distance) are implemented in the model.

The linear-threshold activation function is given by *g*(*x*) = [*x*]^+^ with *g*(*x*) = 0 for *x* ≤ 0 and *g*(*x*) = *x* otherwise. The dynamics of a homogeneous population of neurons with firing-rate vector $f_{j} \left( t \right) = \left[ {s_{j} \left( t \right)} \right]^{ + }$ is described by

1 $$ \dot{s}_{j} \left( t \right) = \left( {b_{j} + \mathop \sum \nolimits W_{i} \cdot s_{i} \left( t \right) + x\left( t \right) - s_{j} \left( t \right)} \right)/\tau $$

with *b*_*j*_ being a bias term, *τ* the time constant, and *x*(*t*) external input. *W*_*i*_ is a matrix of synaptic weights describing the connectivity of the network. The Gaussian synaptic weight between neuron *j* and *k* is given by $w_{jk} = c \cdot e^{{ - \left( {j - k} \right)^{2} /\sigma^{2} }}$ with *c* being a scaling factor and *σ* the width. The resulting Gaussian tuning functions are schematically depicted in Fig. 2 (upper left).

The two populations representing the dorsal tegmental nuclei (DTN) receive angular velocity input *v*_0_ ± *v*(*t*) are connected by mutual inhibition, and inhibit the population representing the lateral mammillary nuclei (LMN). The LMN sends excitatory projections bilaterally back to DTN and forward to the ADN, which in turn projects to PoS (for neural connectivity, see, e.g., [43]). The DTN–LMN connectivity results in a so-called ring attractor. The following equations describe the connectivity of the network (*b* bias, *W* weight matrices):

2a $$ f_{\text{DTNl}} = b_{\text{DTN}} - W_{\text{in}} \cdot s_{\text{DTNr}} \left( t \right) + W_{\text{ex}} \cdot s_{\text{LMN}} \left( t \right) + v_{0} + v\left( t \right) $$

2b $$ f_{\text{DTNr}} = b_{\text{DTN}} - W_{\text{in}} \cdot s_{\text{DTNl}} \left( t \right) + W_{\text{ex}} \cdot s_{\text{LMN}} \left( t \right) + v_{0} - v\left( t \right) $$

2c $$ f_{\text{LMN}} = b_{\text{LMN}} - W_{\text{LMNl}} \cdot s_{\text{DTNl}} \left( t \right) - W_{\text{LMNr}} \cdot_{\text{DTNr}} \left( t \right) $$

2d $$ f_{\text{ADNl}} = b_{\text{ADN}} + W_{\text{ex}} \cdot s_{\text{LMN}} \left( t \right) $$

2e $$ f_{\text{ADNr}} = b_{\text{ADN}} + W_{\text{ex}} \cdot s_{\text{LMN}} \left( t \right) $$

2f $$ f_{\text{PoS}} = b_{\text{PoS}} + W_{\text{ex}} \cdot s_{\text{ADNl}} \left( t \right) + W_{\text{ex}} \cdot s_{\text{ADNr}} \left( t \right). $$

Note that the projections from the two DTN populations to LMN are spatially shifted by a constant angle to accomplish integration of head velocity. The bilateral ADN populations are simulated as separate networks, since there is no evidence for thalamic commissural connections. Finally, the PoS network receives convergent input from both ADN populations and is read out for head direction using standard population decoding.

Simulation of the intact network confirms that it is able to hold a head direction when there is zero velocity input, that is, when it is not rotating (see first and last 1000 time units of Fig. 5b) and that it integrates angular velocity input to a veridical representation of changing head direction (compare the overlapping red and blue traces in Fig. 5b, lower part). For the simulation of lesions, the respective output firing rates of damaged neurons are set to zero (see further description in “Results”).

## Results

### Clinical study

#### Signs and symptoms

Rotational vertigo (*n* = 9, 14%) was rare and reported to occur only as a transient phenomenon, lasting hours or less than 1 day at the onset of the disorder in eight of the nine patients (see Table 1). It was associated with a postural imbalance or unsteadiness in six patients and nausea in four. It was accompanied by an internuclear ophthalmoplegia in four and an upbeat nystagmus in one patient, signs that indicate a lower midbrain or ponto-mesencephalic lesion. The character of vertigo was described as swaying vertigo (*n* = 7; 11%), and unspecific dizziness without a false or distorted sense of body motion (*n* = 7, 11%). The prevailing symptoms of the patients were double vision and blurred vision (*n* = 53, 80%) and imbalance of posture and/or gait (*n* = 23, 36%). At the time of the first neurological examination upon entering our clinic (day 1), only five patients (nos 3, 12, 41, 51, and 63) had a transient horizontal spontaneous nystagmus (detected with Frenzel’s glasses; two leftward, three rightward, three with a rotatory component) which was gone at day 2. One of these five patients complained about initial rotational vertigo (no 12), one about swaying vertigo (no 41), and another one about unspecific dizziness (no 63).

**Table 1:** Nine patients with acute unilateral midbrain infarctions presenting with initial rotational vertigo

Patient (no/gender/age)	Lesion	Side	Symptoms at onset	Ocular motor signs (at day)	Stance/gait	OTR (to R/L)	SVV (°)
9. F27	Ponto-mesencephalic	L	Rot v 2 days, double vision, nausea	(2) INO L, N.III paresis	Unspecific imbalance	Complete R	+ 8°
12. F60	Caudal midbrain	L	Acute rot v < 1 day, double vision, nausea	(1) INO L, rotatory SPN L GEN L, R	Imbalance, falls R	Complete R	+ 10°
17. M46	Midbrain	L	Rot v 1 h, nausea, emesis, double vision, drowsiness	(7) Vertical gaze palsy U/D, CRN, N.IV palsy L	Slight imbalance	Incomplete R	+ 3.6
28. F76	Midbrain thalamus	L	Rot v < 1 day, double vision, headache	(2) Vertical gaze palsy U, N.IV R, Horner R, saccadic pursuit	Imbalance	Incomplete R	+ 8°
33. F47	Paramedian midbrain	L	Rot v, swaying v < 1 day, headache, vertical double vision	(2) Vertical GEN, vertical gaze palsy U/D, CRN, convergence palsy, OKN diminished U/D	Imbalance	Complete R	+ 9.5°
35. F42	Ponto-mesencephalic	L	Rot v, nausea, double vision	(2) Ptosis L, INO L, abduction deficit L	Falls R	Complete R	+ 15°
42. F77	Midbrain	R	Rot v < 1 day, horizontal double vision	(1) INO R, slow saccades U	(Not documented)	Incomplete L	− 9°
48. F63	Midbrain	R	Rot v for seconds during head movements < 1 day, headache, vertical double vision	(5) N.III L, GEN R/L, saccadic pursuit all directions	Normal	Incomplete L	− 7°
49. F82	Midbrain ponto-mesencephalic	R > L	Rot v < 1 day, nausea, vertical double vision	(3) UBN, GEN U/R/L	Imbalance	Incomplete L	− 7.5°

*F* female, *M* male, *L* left, *LE* left eye, *R* right, *R > L* right more than left, *U* up, *D* down, *CRN* convergence retraction nystagmus, *GEN* gaze-evoked nystagmus, *INO* internuclear ophthalmoplegia, *N.III* oculomotor nerve/nucleus, *N.IV* trochlear nerve/nucleus, *OTR* ocular tilt reaction, *rot v* rotational vertigo, *SPN* spontaneous nystagmus, *SVV* subjective visual vertical (tilts in degree; + to the right; − to the left)

Sometimes, a clear differentiation of patients with a swaying vertigo and those with various forms of unspecific dizziness remained uncertain when based solely on the subjective complaints. However, the group with swaying vertigo presented with a postural imbalance, which was multidirectional in four and exhibited a directional preference in three patients (Table 2). In contrast, postural imbalance was found in only one of the seven patients in the group with unspecific dizziness.

**Table 2:** Patients with acute unilateral midbrain infarctions presenting with swaying vertigo (*n* = 7) or unspecific dizziness (*n* = 7)

Patient (no/gender/age)	Lesion	Side	Symptoms at onset	Ocular motor signs (at day)	Stance/gait	OTR (to R/L)	SVV (°)
**Swaying vertigo**							
1. F45	Midbrain paramedian T	R	Acute swaying vertigo, gait imbalance, double vision	(3) Vertical gaze palsy, CRN, OTR L	Imbalance L	Complete L	− 8°
11. M33	Midbrain anteromed. T	L	Acute headache, swaying v, double vision for a few hours	(3) Vertical gaze palsy U/D, UBN, OKN reduction U/D	Falls R	Complete R	+ 10°
18. F56	Paramedian T rostral midbrain	L	Swaying v, double vision	(6) Skew R > L, slow vertical saccades, incomplete N. III palsy L	Diffuse imbalance	Complete R	+ 8°
25. F84	Paramedian midbrain	R	Acute swaying v, double vision	(1) Vertical gaze palsy D > U, CRN, incomplete N.III palsy R	Diffuse imbalance	Incomplete R	+ 1.4°
41. F38	Paramedian midbrain	L ≫ R	Acute swaying v, double vision, nausea	(1) rotatory SPN R, GEN diss. R (3) GEN L/U, fascicular N.IV palsy R, OKN palsy D, pursuit impairment all	Falls backward	Complete R	+ 9.2°
52. M75	Paramedian midbrain	R	Acute swaying v to L, ptosis R	(1) N. III palsy R with ptosis, saccade palsy and slowing U, pursuit impairment all directions	Falls L	Complete R	+ 10°
62. M57	Paramedian midbrain	R	Acute swaying v, vertical double vision, occipital headache	(2) Mild fascicular N.III palsy R, skew L > R, GEN all directions, saccadic slowing U/D	Mild diffuse imbalance	Incomplete L	− 8°
**Unspecific dizziness**							
2. F73	Paramedian midbrain	L	Numbness of the head, imbalance R, dysarthria	(1) N.III L with ptosis, INO L, GEN U/D, gaze palsy U/D, pursuit deficits all d	Falls R	Complete R	+ 18°
10. F76	Midbrain R > L thalamus R	R ≫ L	Acute dizziness, transient double vision, dysarthria	(4) Skew R > L, vertical gaze palsy U/D, CRN, slow vertical saccades	No imbalance	No	No
20. M37	Thalamus midbrain	L	Acute numbness, double vision, mild headache	(2) N. III L, CRN, GEN D, slow saccades D, gaze palsy U, exophthalmus L	No imbalance	Incomplete R	+ 8°
32. M70	Anteromed. midbrain to T	L	Acute transient dizziness, double vision, dysarthria, disorientation	(5) Gaze palsy U, no SPN, pursuit deficits all directions, slow vertical saccades	No imbalance	Incomplete R	+ 16°
58. M26	Tectum of midbrain	L	Acute dizziness, double vision with upward gaze	(2) Ptosis L, LE: partial gaze palsy U	No imbalance	Not clear	+ 3.2°
61. F51	Tegmentum of midbrain	L	Acute dizziness, 7 h later double vision	(2) Fascicular N.III L, skew L > R, UBN head tilt to R, GEN R/L/U, pursuit deficits all directions	No imbalance	Complete R	+ 6.6°
63. F49	Anterior T to midbrain	L	Acute dizziness, hemiparesis R, dysarthria, double vision	(2) Gaze palsy U, SPN L, skew L > R CRN, OKN U lost D diminished, slow saccades U > D	Diffuse imbalance	Complete R	+ 13°

*F* female, *M* male, *L* left, *LE* left eye, *R* right, *U* up, *D* down, *CRN* convergence retraction nystagmus, *GEN* gaze-evoked nystagmus, *INO* internuclear ophthalmoplegia, *N.III* oculomotor nerve, *N.IV* trochlear nerve, *OTR* ocular tilt reaction, *SPN* spontaneous nystagmus, *SVV* subjective visual vertical (tilts in degree; + to the right; − to the left), *T* thalamus, *v* vertigo, *R ≫ L* right much more than left, *skew R > L* vertical divergence of the eyes with the right eye over the left eye

A fourth group included 20 patients with clinical signs of non-paretic postural imbalance or unsteadiness without complaints of vertigo or dizziness. This imbalance exhibited a directional preference in 11 (7 to the right and 4 to the left) and was diffuse in nine patients.

In addition to the above-described clinical features, it was striking that the most frequent vestibular signs were not those in the yaw plane (i.e., horizontal semicircular canal function, the main focus of our study) but rather those in the “graviceptive” roll plane (otolith and vertical semicircular canal function). These “static” signs of a vestibular tone imbalance in the roll plane were deviations of the SVV (*n* = 56, 89%), vertical divergence of the eyes (skew deviation, *n* = 51, 81%), and an incomplete or complete OTR (*n* = 46, 73%). In all of these patients, the head, ocular motor, and perceptual tilts were directed contralaterally to the mesencephalic lesion side: rightward in left-sided lesions and leftward in right-sided lesions.

Neuroorthoptic findings are depicted for the patients presenting with vertigo or dizziness in Table 1 (rotatory vertigo) and Table 2 (swaying vertigo and unspecific dizziness). Both tables also contain the assumed level of the meso-diencephalic stroke lesion based on a combination of the clinical and imaging findings.

**Fig. 3:**
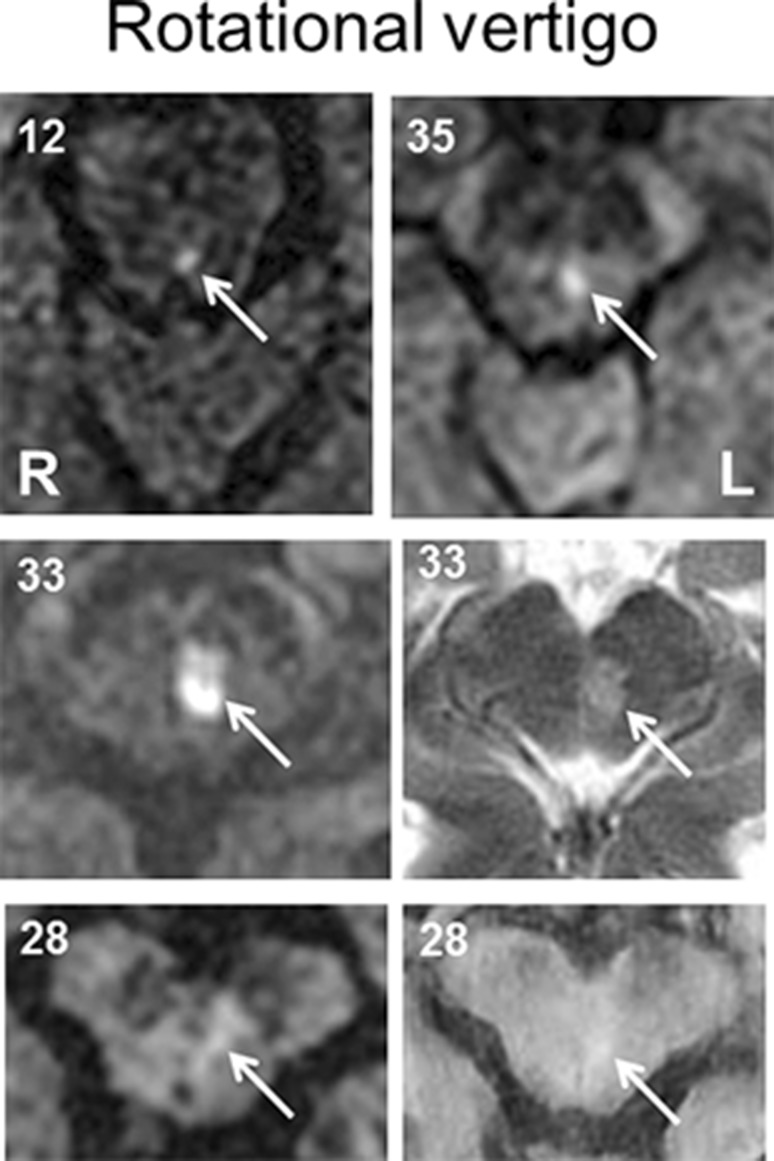
MRI scans of four patients (nos. 12, 28, 33, and 35) who manifested with acute transient rotational vertigo due to circumscribed unilateral midbrain strokes (diffusion- and T2-weighted sequences). In the upper panel two transversal sections of the caudal midbrain (no. 35; with left internuclear ophthalmoplegia, INO) and the midbrain close to the pons (no. 12, with left INO) are depicted with small unilateral infarctions of the left tegmentum affecting the medial longitudinal fascicle which contains ascending vestibular fibers. In the other two patients (no. 33 with vertical gaze palsy and convergence retraction nystagmus, and no. 28 with vertical gaze palsy and fascicular fourth nerve palsy right) the unilateral strokes involve a midbrain level close to the oculomotor nucleus, the interstitial nucleus of Cajal, and the rostral interstitial nucleus of the MLF, see also Table 1

**Fig. 4:**
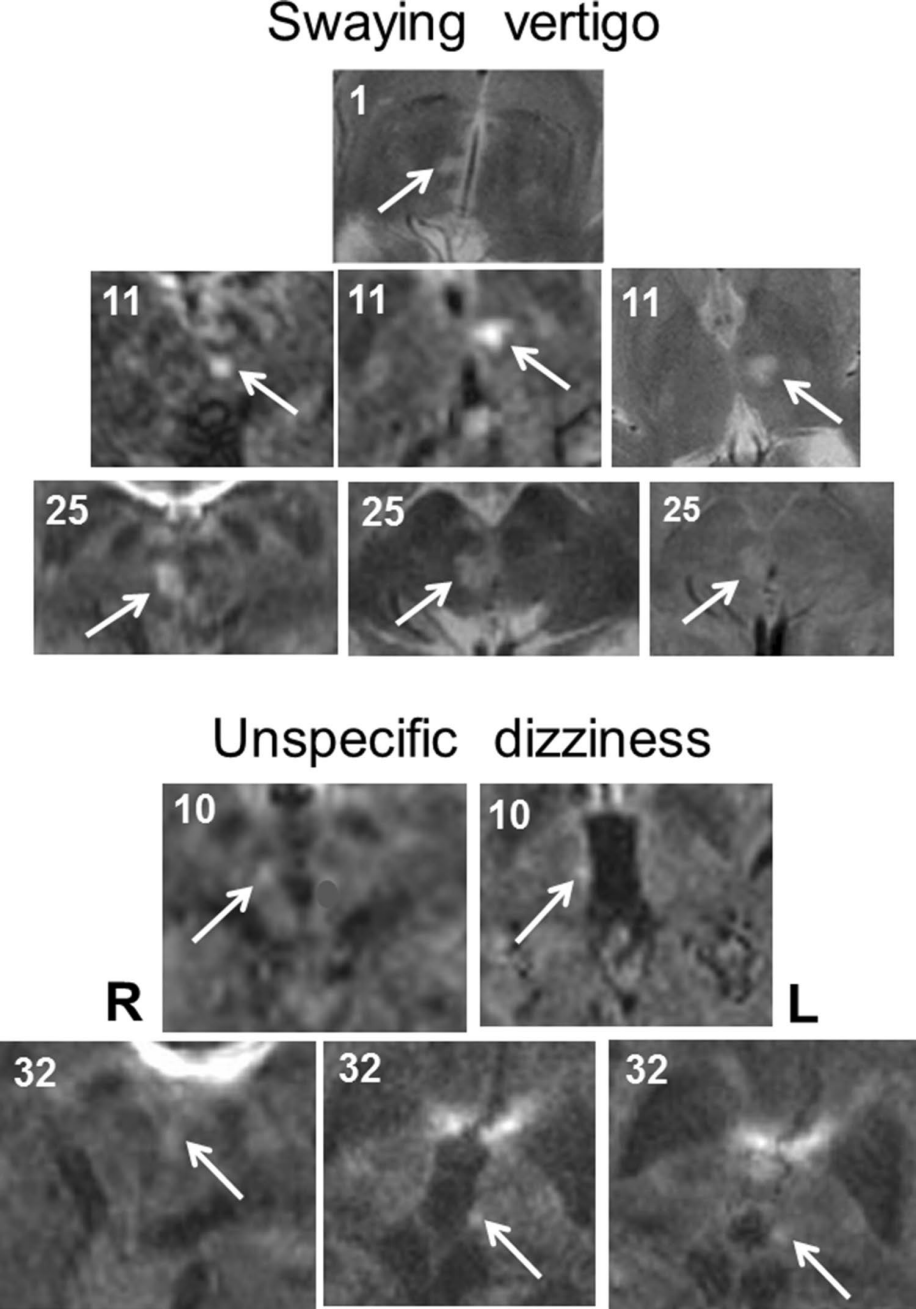
Transverse sections of MRI scans of five patients (nos. 1, 10, 11, 25, and 32) who manifested with acute swaying vertigo (upper panel: nos. 1, 11, and 25) or unspecific dizziness (lower panels: nos. 10 and 32) (diffusion- and T2-weighted sequences). In these patients, the unilateral midbrain strokes affected more rostral structures including the oculomotor nucleus, the interstitial nucleus of Cajal, and the rostral interstitial nucleus of the MLF (nos. 11 and 25) or meso-diencephalic structures up to the paramedian thalamus (nos. 1, 10, and 32), see also Table 2. *MLF* medial longitudinal fascicle

MRI examples of typical cases with rotational vertigo, swaying vertigo, and unspecific dizziness are given in Figs. 3 and 4. Most of the patients had very small stroke lesions. In the patients with rotational vertigo the lesions appeared to be located in the lower paramedian midbrain and at the ponto-mesencephalic level, whereas the lesions in the patients with swaying vertigo and unspecific dizziness were more often in the upper paramedian midbrain and at the meso-diencephalic level, some extending into the centromedian thalamus.

Non-vestibular signs and symptoms were headache, which occurred in eight of the 63 patients (12%), three of whom belonged to the group with transient rotational vertigo. Dysarthrophonia was found in three patients; none had vertigo, dizziness, or postural imbalance.

### Network simulation

#### Effects of a unilateral peripheral or brainstem lesion

When firing of one side is completely abolished in a network with a push–pull organization (as present in the brainstem), the immediate functional consequence will be equivalent to that of a maximally inhibiting input to the target neurons, since both lesion and maximum inhibition will result in zero input. The most likely immediate interpretation will thus be that of a stimulus causing maximum inhibition. For a group of neuron coding for angular velocity, this interpretation means fast rotation in the off-direction, e.g., rotation to the right for a lesion on the left side (assuming ipsilateral on-direction). Such an interpretation of the afferent input will cause severe vertigo, as observed in peripheral lesions of the vestibular nerve. Our computational simulation of a peripheral lesion confirms this by leading to a continuous rotation of the bump of activity in the head direction cell network even without external input (not shown, but indistinguishable from Fig. 6a). A unilateral lesion of the network at the level that forms the transition point between head velocity and head direction cells (the DTN) still results in a continuous rotation of the hill of activity at higher levels (Fig. 6a), which can be interpreted as vertigo.

#### Effects of a unilateral lesion in the head direction cell network

In the following, we assume that neurons coding for head direction is randomly distributed over the two sides of the respective nuclei with no specific preference, but that they together form a population via commissural connections. For a unilateral lesion, we thus simply set the firing rate of about 50% of all neurons to zero. Simulation of lesions at the LMN stage of the network shows that unilateral damage does not cause a continuous motion of the hill of activity (Fig. 6b). The immediate consequence of the unilateral lesion at the level of the LMN is a slight random bias, which may manifest as a direction-unspecific deviation of the straight ahead, together with an increased sensitivity, that is, an overestimation of rotation. In addition, the rotational velocity of the hill of activity no longer faithfully mirrors the imposed angular velocity, but slightly oscillates around it. This would lead to a discrepancy between visually perceived rotation (assuming that visual areas for motion perception are intact) and vestibular-mediated change in head direction, which may be interpreted as a to-and-fro wobbling motion specifically while turning.

The next topographic stage within the vestibular circuit, the thalamic ADN, is assumed to be organized bilaterally as two separate networks, both of which provide convergent input to the final PoS stage. Unilateral lesion of the ADN thus simply removes one of the two redundant sources of input for PoS. As a result, the functional consequence of complete inhibition of one side of the ADN is much less obvious (Fig. 6c). According to our simulations, the main effect is a strong reduction of head direction cell firing in the PoS: there are no other effects (see black arrows in Fig. 6c). However, depending on the exact strength of the network connections, a unilateral ADN lesion may also completely abolish head direction cell firing in the PoS. Finally, the effects of a lesion of the PoS depend again on its intrinsic connectivity and the read-out stages above. Without recurrent connectivity, a unilateral PoS lesion would reduce final read-out strength, possibly also causing effects like wobbling during turns similar to those obtained for a unilateral LMN lesion.

## Discussion

### Clinical manifestations of unilateral vestibular midbrain syndromes

The major finding of this retrospective clinical study was that only 14% of the patients with acute unilateral midbrain strokes exhibited rotational vertigo, which was transient, lasting less than one day. In only three a transient spontaneous nystagmus on the day of admission was seen. From days 3 to 8 after the onset of the disorder—when rotational vertigo had ceased—no spontaneous vestibular nystagmus could be observed in the neuroorthoptic analysis. The low frequency of rotational vertigo, its transient character, and the rarity of spontaneous nystagmus make a unilateral vestibular lesion at midbrain level different from those syndromes that occur with more caudal lesions of the bilateral central vestibular network in the brainstem. Rotational vertigo is typical for unilateral lesions at the root entry zone of the eighth nerve, the medial and superior vestibular nuclei, the nucleus prepositus hypoglossi, the cerebellar peduncles, cerebellar vermis (uvula and nodulus), and cerebellar tonsil and flocculus (reviews: [8, 9, 31]). All the latter may mimic an acute unilateral peripheral vestibulopathy as it occurs with vestibular neuritis and, therefore, was also called ‘vestibular pseudoneuritis’ [14, 15]. A three-step bedside test (HINTS)—head impulse test, nystagmus, and skew deviation—can detect this central variant of an acute vestibular syndrome with a sensitivity of more than 90%, which is superior to early diffusion-weighted MRI sequences [12, 16].

In the current study, an exact localization of the causative midbrain lesions in our nine patients who initially had rotational vertigo was only possible in four, because MRI failed to detect the very small lesions or the rostro-caudal extent included larger parts of the midbrain tegmentum in the other patients (Fig. 3). In the groups with swaying vertigo and unspecific dizziness, upper midbrain lesions involved the meso-diencephalic structures up to the thalamus (Fig. 4). However, the fact that four of the patients with initial rotational vertigo had an additional internuclear ophthalmoplegia and one an upbeat nystagmus helped to localize the lesions to the more caudal part of the midbrain tegmentum. The caudal extent could be inferred from the directions of SVV tilts, skew deviation, and OTR which were contralateral to the side of the lesions. This indicated that the lesions were above the level of the pontine crossing of graviceptive pathways, thus superior to the vestibular nuclei [21, 23, 39, 50, 51]. Thus, rotational vertigo rarely occurs with lesions above this level. If the symptomatology of the patients with rotational vertigo is attributed to a specific motion-detecting cell system, it is most likely that they suffered from a transient dysfunction of the head-angular velocity cell system supplied with horizontal semicircular canal function.

The patient group 2 with swaying vertigo and group 3 with unspecific dizziness could reflect a disturbance of another cell system, namely, that mediating head direction and orientation in space. The cooperation between angular velocity and head direction signals for the perception of motion is similar to the ocular motor integration from velocity to position, which is mediated in the pons for eye–head coordination in the horizontal yaw plane and in the midbrain tegmentum at the level of the interstitial nucleus of Cajal for eye–head coordination in the in vertical roll and pitch planes. An integration of perceived angular velocity into position, i.e., the actual head direction in space, is the basis for a continuous updating of the internal model or cognitive map of the individual’s position in the environment [52]. Hence, the symptoms of a dysfunction of the head direction cell system should not be a perceived rotational vertigo but more likely a motion-induced swaying vertigo with directional disorientation resulting in a postural imbalance and unsteadiness. Therefore, the patients of group 4 who exhibited no subjective dizziness but instead a uni- or multidirectional imbalance would also fit into the concept of an impaired function of the head direction cell system. However, the drawback of such a one-sided interpretation is, namely, that it ignores the high frequency of an associated vestibular tone imbalance in the roll plane, which was found in up to 89% of all 63 patients with unilateral midbrain strokes. This graviceptive tone imbalance manifested in perceptual tilts of verticality as well as ocular motor and head tilts (ocular tilt reaction). Body lateropulsion may rarely occur in thalamic astasia and more frequently in medullary brainstem syndromes (e.g., Wallenberg syndrome); however, it was not a feature reported in the patients enrolled in our study. In the model we focused on rotational vertigo in the horizontal plane and, therefore, on semicircular canal function.

### Mathematical modelling of unilateral vestibular syndromes in yaw plane

To mathematically model the clinical syndromes of a disturbance of unilateral horizontal semicircular canal function or unilateral lesion of the network at subsequent levels, we implemented an extended version of a previously published neural network model of the head velocity and head direction cell circuitry. As we have shown, the model can simulate the differential effects of unilateral central vestibular lesions of the caudal and rostral brainstem with respect to the occurrence of rotational vertigo. Our simulation results also agree with findings in animal experiments, which have shown that head direction cell activity is disrupted after occlusion of the semicircular canals in the chinchilla [28] and in the epistatic circler mice with bilateral malformation of the horizontal semicircular canals [30]. Furthermore, in rodents, a unilateral lesion of the lateral mammillary nucleus did not impair firing of head direction cells in the next stage, the antero-dorsal nuclei of the thalamus [53].

Our model is based on neuroanatomical and neurophysiological findings. Most importantly, as summarized by Taube and co-workers [43], the distribution of head direction and head-velocity cell systems is such that the head-angular velocity cell system is primarily located in the caudal brainstem, whereas the head direction cell system increasingly dominates over the head-angular velocity system at higher mesencephalic and thalamic levels. The other important fact used in the model is that head-velocity cells, for example, in the vestibular nuclei, show a clear side-specific preference in coding angular velocity, while no such difference in directional coding or preferred coding direction has been found in head direction cells on both sides of the brain.

**Fig. 5:**
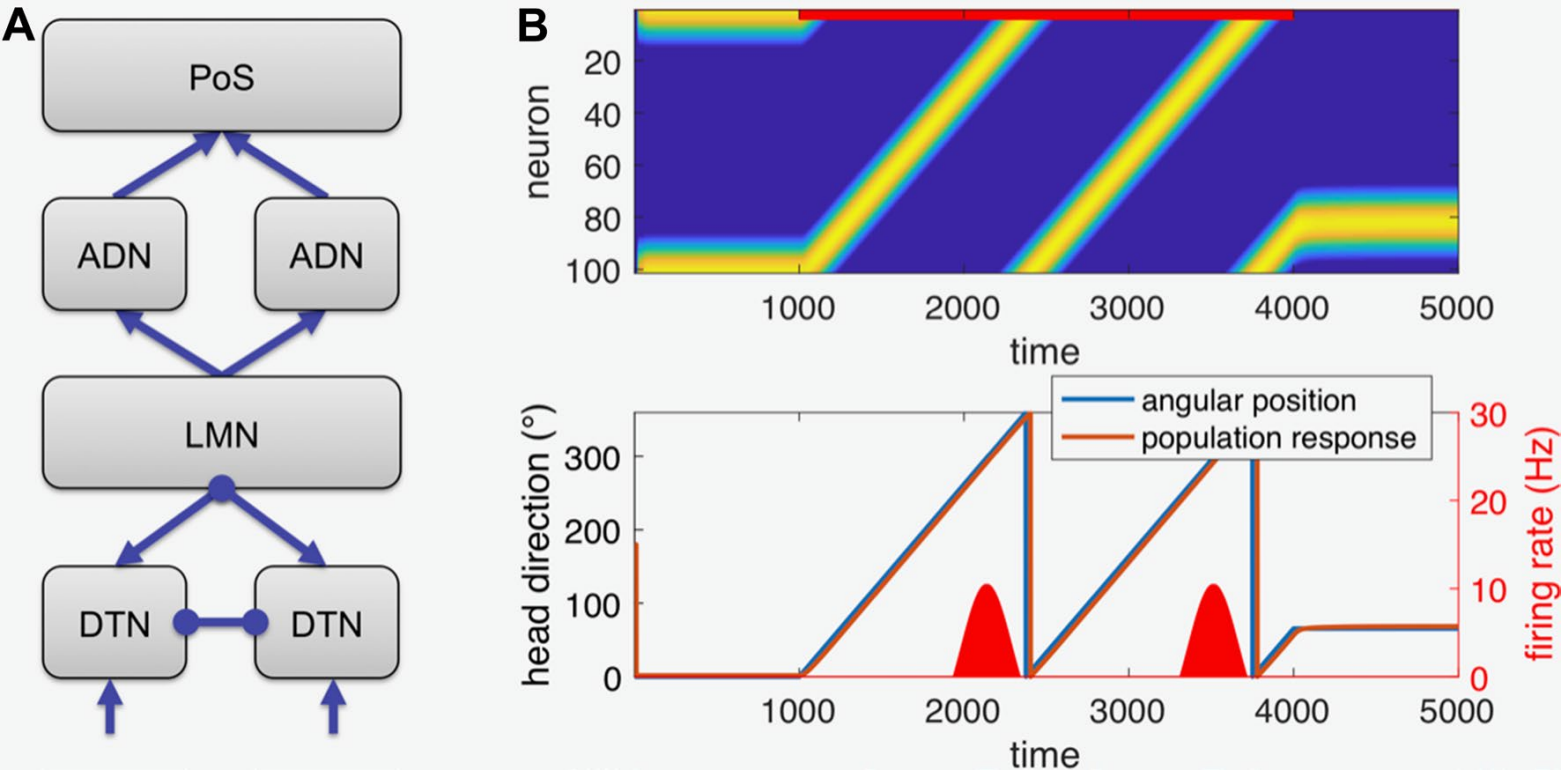
Simulation of head direction cell population. **a** Schematic diagram of the network: the dorsal tegmental nuclei (DTN) receive angular velocity input and are mutually connected by inhibitory commissures (inhibition is indicated by circular line endings). The DTN project an inhibitory connection to the lateral mammillary nuclei (LMN) and receive an excitatory connection from the LMN (indicated by arrowheads). LMN projects to the thalamus (antero-dorsal nuclei, ADN), which in turn projects to the postsubiculum (PoS). The PoS provides the read-out stage for the model. **b** Normal function for a stimulus of 1000 time units of constant angular position at 0° followed by 3000 time units of continuous turning, and another 1000 time points of constant position at 65°. Upper part shows the color-coded firing rate of the 100 simulated neurons in PoS plotted over time (yellow color denotes maximum firing rate, blue color is zero firing rate). The neurons are sorted by preferred direction, so that the yellow trace depicting the moving bump of neural activity faithfully tracks the true rotation. Lower part plots the true head direction (blue) and the decoded population response derived from the firing of the neurons in the upper part over time. Both true angular position and decoded population response overlap indicating that the network faithfully represents angular head direction and implements mathematical integration of angular velocity over time to head direction. The lower red bumps (corresponding to the axis at the right) show the activity of a single-neuron coding approximately for a head direction of 300° with a maximum firing rate of 10 Hz. The lower part of **b** is constructed after corresponding figures from the literature such as Fig. 3a from [30], to which it can be directly compared

**Fig. 6:**
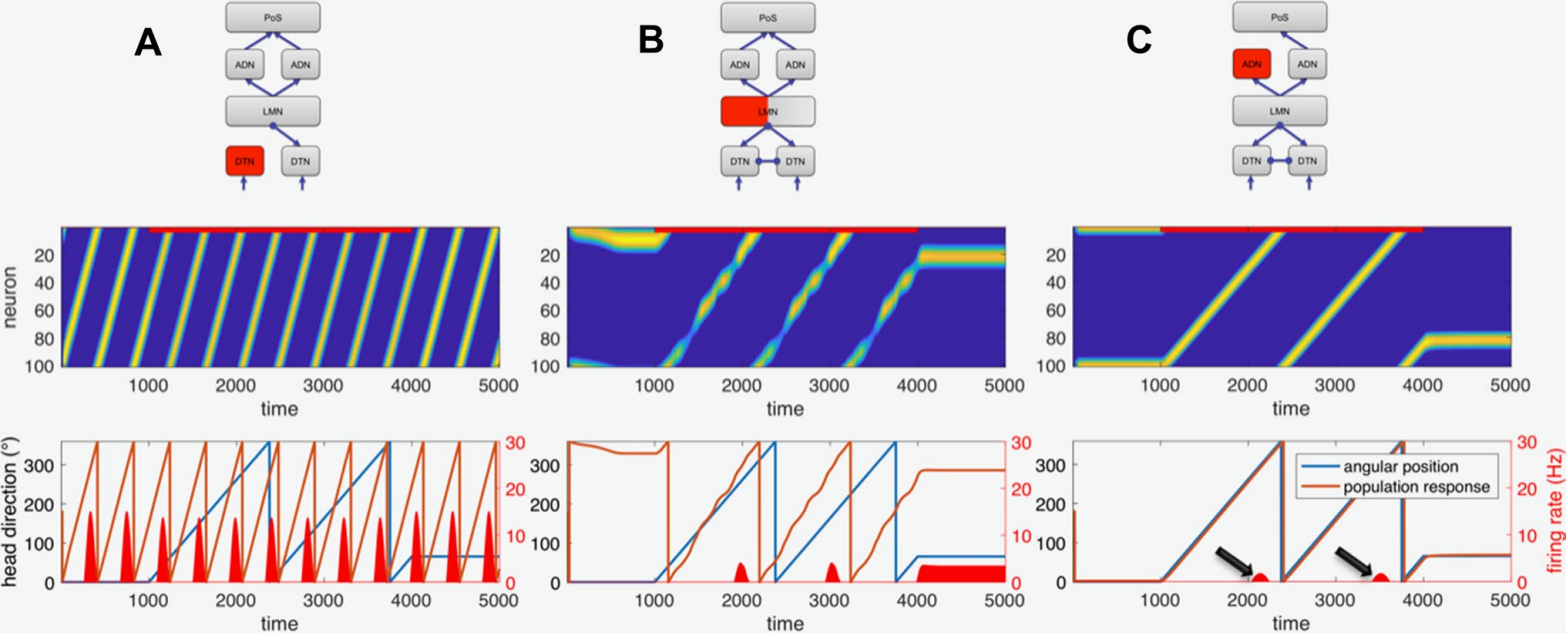
Simulation of unilateral lesions in the network. The schematic depiction in the first row indicates the location of the lesion by red color and missing connections (compare to Fig. 5a). The second and third rows show network activity and decoded head direction as in Fig. 5b. **a** Unilateral lesion of the network at the angular velocity level (DTN or below) leading to continuous turning (vertigo) even when there is no stimulus (constant positions). **b** Lesion at the LMN level of the network involving head direction cells. Approx. 50% of the neurons have been silenced to simulate a unilateral lesion. The network shows a steady population response for the periods of constant position, which, however, deviates from the true position (possibly indicating a deviation of the straight-ahead position). During the stimulation, the network response is irregular and unstable (note the wobbly response trace) and indicates faster turning compared to the stimulus. **c** Unilateral lesion at the ADN level of the network. Due to the assumed redundant coding in both parts of the ADN the effect of a unilateral lesion is almost negligible except for strong decrease in amplitude of the PoS neurons (see black arrows pointing to small red bumps for the firing of the neuron tuned to approx. 300°, compare with large bumps in Fig. 5b). The population response (lower part, red) follows the true head direction. Note, however, that slightly different network parameters might cause abolishment of PoS head direction cell activity

In the model, a lesion of a population of neurons is simulated by setting the firing rates of these neurons to zero. The functional effect of the lesion strongly depends on the type of population (excitatory or inhibitory), but also on its connectivity. Complete unilateral ablation of a network with population coding such as the head direction cell network can have different types of functional consequence. For example, if the population codes for spatial position in a topographic map-like fashion as is the case for the primary visual areas, then unilateral removal of this area will lead to cortical blindness in that hemifield (see also [54]). However, the population-coding network of the head direction cell system is not organized topographically in such a map-like layout. Instead neurons on both sides of the brain can code for similar head direction (see Fig. 2, upper right side). Interaction between populations on both sides happens via commissural connections (at the level of the LMN), so that both populations together form the network. As we have shown, unilateral lesion of such a network does not cause continuous rotation (Figs. 5, 6).

Similarly, when both sides do not interact via commissural fibers (as in the case of ADN), ablation of one side will not cause continuous rotation. In coding schemes like those probably used by the head direction cell system, the amplitude of the population firing rate is thought to inversely code for the uncertainty of the variable it represents. A larger uncertainty will lead to less overall firing and vice versa. Thus, the functional meaning of a complete lesion would be maximal uncertainty about which value of the variable is currently encoded. In other words, the lesioned population of the head direction cell system would indicate that nothing is known about the current head direction. With a unilateral lesion, this uncertainty would be partly compensated by the intact contralateral population, so that the net effect would only be a decrease in certainty about the current head direction. Such a decrease in certainty might be detected by psychophysical testing of just-noticeable differences, perceptual thresholds, or intra-individual variability in spatial tasks. Such clinical tests are not yet available and have to be developed.

### Limitations

The clinical data are based on a retrospective analysis, because we aimed to determine the percentage of rotational vertigo in unilateral midbrain lesions in a larger patient group. Therefore, clinical data were analysed from a period of 10 years. This allowed a comparison of the effects of upper versus lower brainstem lesions. However, the retrospective character and the limitations of the MRI made it impossible to attribute the particular lesions to single nuclei as defined in the animal studies (Fig. 2). Another limitation is that measurements of heading (e.g., subjective straight ahead) had not been made. However, according to the spontaneous patients’ reports, unilateral upper midbrain and thalamic lesions often caused a direction-changing unsteadiness and imbalance of gait without any clear directional preference, which is in line with our model simulations.

A limitation of our first model approach is that it is restricted to the head direction cell system and focuses on horizontal semicircular canal input. There are animal data which showed that cells in the LMN (Fig. 2) not only fire as a function of angular head velocity in the yaw plane but also in the pitch plane [55] and that angular head-velocity modulates activity of head direction cells in ADN [56–58]. The model neglects input from the vertical semicircular canals and the otoliths. Indeed, the clinical data exhibited an involvement of graviceptive vestibular function in the roll plane in the majority of the patients. Graviceptive vestibular function in the roll plane was not included in the current model, because up to now, quantitative animal data on otolith und vertical semicircular canal function are sparse, with the exception of a recent macaque study by Laurens and co-workers [59] on the gravity orientation tuning in anterior thalamic neurons. Further modelling of graviceptive function is required, because human studies on patients with unilateral central vestibular lesions—from the vestibular nuclei along the ascending pathways via the medial longitudinal fascicle to the eye–head coordination centres in the rostral midbrain, the vestibular thalamus, and the vestibular cortical network—all result in directional tilts in the roll plane [39]. The clinical manifestation of a graviceptive tone imbalance mainly involves perception of verticality in thalamic and vestibular cortex lesions and a combination of perceptual, ocular motor, and head tilts in caudal brainstem lesions. Thus, static vestibular function in the roll plane differs from dynamic vestibular semicircular canal function in the yaw plane as discussed above. However, the static effect might still be due to a coding similar to the head direction system, which was recently described in the thalamus of monkeys [59] and was implemented in modelling tilts of perceived verticality in the roll plane due to unilateral vestibular lesions from the labyrinth to the cortex [60].

## Conclusions

The clinical data on unilateral lesions induced by acute midbrain strokes that affect the bilaterally organized vestibular system are compatible with the different distribution of the two separate cell systems that mediate angular motion in space. A unilateral dysfunction of the angular head-velocity cell system located at the lower brainstem and cerebellar level results in rotational vertigo; a unilateral dysfunction of the head direction cell system located at the upper brainstem, thalamus, and cortical level often results in no apparent vertigo symptoms or swaying vertigo with directional spatial disorientation and unsteadiness. Thus, the described attractor model was able to predict and confirm our clinical findings of the differential effects of unilateral caudal and rostral brainstem lesions on vestibular function and its disorders in the yaw plane. The functional significance of the two cell systems relies on a transition from head velocity to head direction, as a precondition for continuous updating of body orientation in space and for adequate navigation within the environment for an individual in motion.

## Abbreviations

AVS: Acute vestibular syndrome
OTR: Ocular tilt reaction
SVV: Subjective visual vertical
PoS: Postsubiculum
ADN: Antero-dorsal nuclei of the thalamus
DTN: Dorsal tegmental nucleus
LMN: Lateral mammillary nucleus
INC: Interstitial nucleus of Cajal
MLF: Medial longitudinal fascicle
riMLF: Rostral interstitial nucleus of the MLF
